# Testicular torsion: its effect on autoimmunisation, pituitary–testis axis and correlation with primary gonadal dysfunction in boys

**DOI:** 10.1038/s41390-021-01382-0

**Published:** 2021-02-18

**Authors:** Paweł Osemlak, Konrad Miszczuk, Grzegorz Jędrzejewski, Paweł Nachulewicz, Iwona Beń-Skowronek, Agnieszka Brzozowska

**Affiliations:** 1 The Chair and Department of Paediatric Surgery and Traumatology, Medical University of Lublin, Lublin, Poland; 2 The Department of Paediatric Endocrinology and Diabetology with Endocrine-Metabolic Laboratory, Medical University of Lublin, Lublin, Poland; 3 The Department of Paediatric Radiology, Medical University of Lublin, Lublin, Poland; 4 Department of Medical Informatics and Statistics with E-learning Lab, Medical University of Lublin, Lublin, Poland

## Abstract

**Background:**

Torsion of the testis is an urgent surgical condition that endangers the viability of the gonad and the fertility of the patient. Our aim was to assess potential autoimmune processes and hormonal abnormalities in boys operated on due to that illness.

**Methods:**

The authors evaluated the levels of antibodies against sperm and Leydig cells, concentrations of follicle-stimulating, luteinizing and anti-Müllerian hormone, testosterone, oestradiol and vascular endothelial growth factor in the serum in 28 boys operated on due to torsion of the testis. Patients’ sexual maturity was assessed according the Tanner scale (group G1, G4 and G5).

**Results:**

No antibodies against sperm or Leydig cells were found in the serum. Statistically significant differences in follicle-stimulating and anti-Müllerian hormone concentrations were observed in the G1, and they were higher in the study than in the control group. There were no statistically significant differences in luteinizing hormone, testosterone, oestradiol and vascular endothelial growth factor concentrations in the study group or control group. Testosterone concentration was unrelated to total testicular volume.

**Conclusions:**

Results did not confirm the autoimmune process in boys with torsion of the testis. The pituitary–testis axis seems to have sufficient compensation capabilities. However, study results suggest that primary gonadal dysfunction may predispose to torsion.

**Impact:**

- Significant differences exist between the literature data and own results on the formation of antibodies and hormonal changes due to testicular torsion in boys.
- It is a novel, prospective study on antibodies against sperms and Leydig cells in the serum and on hormonal processes occurring as a result of the testicular torsion from the prenatal period to the adolescence with division into pubertal groups.
- The study has revealed sufficient compensation capabilities of the pituitary–testis axis and no autoimmune process in boys with torsion of the testis.

## Introduction

Torsion of the testis (TT) is an urgent surgical condition involving incomplete or complete and single or multiple rotation(s) of the spermatic cord around a long axis with impaired blood flow through the testis.^1,2^ It is the second most common cause of “acute scrotum” conditions in boys after torsion of the appendix testis.^3,4^ According to Arap et al.^5^ and Lee et al.,^6^ the incidence of TT in the world’s male population under 25 years of age ranges from 3 to 25/100,000. The disease mainly affects boys in the neonatal–infant age and at puberty.^2,4,6,7^

Various anatomical abnormalities predispose to TT, such as incorrect attachment of the testis or epididymis in the scrotum, bell clapper deformity (horizontal position of the testis with high attachment of the tunica vaginalis) and anomalies of the gubernaculum of the testis.^6,8–10^ This urgent surgical condition can be caused by injury to the scrotum, intense physical effort or strong spasm of the cremaster muscle.^8^

Rybkiewicz et al.,^7^ Arap et al.^5^ and Lee et al.^6^ report that the operation for detorsion of the testis provides an opportunity to maintain the viability of the gonad within 6 h of the beginning of the torsion, assuming that it is complete (360° or more). If the torsion is not complete, this time is extended. According to research by Yang, 2 h after the beginning of the torsion, a haemorrhagic infarction of the testis develops, irreversible changes in the parenchyma occur after 6 h and total necrosis is seen after 24 h.^2^

There are three different theories of disturbances of function of the testis after torsion and therefore spermatogenesis. The first theory is that testes have features of primary dysplasia with disordered spermatogenesis and abnormal fixation in the scrotum, which predisposes them to turn.^5,11–13^ The second theory is that antibodies against sperm and testicular antigens are formed due to torsion in the serum and semen.^2,5,7,9,14–17^ The third theory is that twisted testis is damaged, including reproductive cells, by ischaemia–reperfusion syndrome, causing oxidative stress.^2,5,9^ Chakraborty^9^ also described the possibility of ischaemic lesions and neurotransmitter dysfunction in the contralateral healthy testis in response to pathological processes in the twisted testis.

TT also causes abnormal functioning of the endocrine pituitary–testes axis. In previous studies on patients after TT, the endocrine function of this axis was within the laboratory norms.^5,18–20^ Follicle-stimulating hormone (FSH), however, showed a tendency towards higher values and testosterone to normal or lower.

Vascular endothelial growth factor (VEGF) is one of the factors that are associated with autoimmune processes. This factor is necessary for the proper development of the testis. Its concentration is temporarily elevated in the case of transient tissue ischaemia and then returns to normal. In the case of an active autoimmune process, its level is sometimes constantly increased.^21–25^

Objectives of this study were: (1) assessment of potential autoimmune processes induced by TT and (2) determining of possible hormonal abnormalities in boys operated on due to TT.

## Methods

### Subjects

From 2012 to 2015, 80 boys were operated on at our department due to TT. They lived in the central-eastern part of Poland. To form the study group, we selected 28 boys, who were subjected to at least 1 year (usually 3 years) of ambulatory observation.

No diseases with a hormonal nor immunological background were reported in patients and their families. The age of boys at the time of surgery ranged from 1 day of life to 15 years. In 15 cases, the testis was detorted and preserved, but it was excised due to necrosis in 13 cases.

Informed consent for the research was obtained from parents or legal guardians of boys and also from patients over 16 years of age due to the legal regulations in Poland.

The research was approved by the Bioethics Commission of the Medical University of Lublin, Poland, No. KE-0254/11/2017.

### Laboratory tests

Patients from the study group gave a 4.9 ml venous blood sample at 11:00, from which, after centrifugation, 2 ml of serum was obtained. The hour of collection was determined by logistic factors in the hospital. The serum has been subjected to the following laboratory tests.

#### Immunological evaluation

The level of antibodies against sperm and antibodies against Leydig cells in the serum was determined. The indirect immunofluorescence technique and conjugates of IgG, IgA and IgM antibodies were applied (Euroimmun, Germany).

#### Hormonal evaluation

The levels of FSH, luteinizing hormone (LH), testosterone and oestradiol were determined using the chemiluminescence method (Siemens, Germany). The levels of anti-Müllerian hormone (AMH) were determined by the immunoelectrochemiluminescence method (Roche, Austria). In order to determine AMH, due to the weakness of the method, we often had to use dilutions of the sample.

#### Evaluation of growth factor

Levels of VEGF were determined using Quantikine ELISA (R&D system and Biotechne Brand, Minnesota, Minneapolis).

The control group for the above determinations consisted of eight boys with normal sexual development, without health problems that could affect the levels of the tested hormones or growth factors, classified according to age in the amount proportional to the study group. These patients were hospitalised in the Paediatric Surgery Clinic of our hospital.

### Puberty groups

For the purpose of comparative analysis, boys from the study group and control group were divided into three puberty groups: pre-pubertal G1 (stage 1 of puberty according to the Tanner scale), pubertal G4 (stage 4 of puberty according to the Tanner scale) and pubertal G5 (stage 5 of puberty according to the Tanner scale). Puberty of the patient was determined at the time of medical examination for this study.

### Study group

We have analysed 28 patients. The G1 group consisted of 11 patients, G4 group included 9 patients and G5 group 7 patients. One patient with stage 3 of puberty according to the Tanner scale was excluded from the analysis for statistical reasons.

### Control group

The pre-pubertal G1 control group consisted of four patients. Each puberty group (G4 and G5) consisted of two patients. Our pubertal grouping was designed to enable comparative analysis of hormonal profiles that had changed with degree of puberty.

### Ultrasound exam

All patients from the study group underwent an ultrasound. Ultrasound examination of the testes was conducted with a Siemens Acuson S2000 apparatus with linear transducer 9–12 MHz. The length, width and thickness of the testis were measured, and then its volume was calculated (length × width × thickness in cm × 0.52).^26^ The statistical relationship between total testicular volume (TTV) and testosterone concentration was determined in patients from the G4 and G5 groups. TTV means the summed volume of both testes (detorted and the contralateral) or only of the contralateral one in case of orchiectomy.

### Statistical analysis

The achieved results were statistically analysed in the Department of Medical Informatics and Statistics with E-learning Lab of our university.

The values of measurable parameters were characterised by the range of values, arithmetic mean (*X*), standard deviation (SD) and median (Me). The normality of the distribution of the analysed parameters was evaluated using the Shapiro–Wilk test.

The *U* Mann–Whitney test (M–W test; *Z*-value) was used to compare two independent groups. The Kruskal–Wallis test (K–W test; *H-*value) was used to compare many groups. The Spearman’s *R* correlation coefficient was used to assess the relationship between independent parameters. A significance level of *p* < 0.05 was set, indicating statistically significant differences or dependencies were accepted. The statistical analysis was conducted using the Statistica 13.0 software (StatSoft, Poland).

## Results

No antibodies against sperm nor Leydig cells were found in the serum of patients from the study and control groups.

The level of FSH in group G1 was statistically significantly higher in the study group than in the control group (*p* = 0.005), while no significant differences between the study and control groups were found in groups G4 and G5 (*p* > 0.05) (Table 1).

**Table 1:** FSH in the study group (in general) and control group—concentration mIU/ml.

Group	Range of values	*X*	SD	Me
Pre-pubertal G1				
Study	0.53–2.15	1.00	0.53	0.76
Control	0.41–0.47	0.45	0.03	0.46
M–W: *Z* = 2.81; *p* = 0.005				
Pubertal G4				
Study	0.81–15.34	6.46	4.50	4.62
Control	1.59–8.71	5.15	5.03	5.15
M–W: *Z* = 0.12; *p* = 0.91				
Pubertal G5				
Study	1.86–10.60	5.04	3.30	4.07
Control	0.95–3.20	2.06	1.56	2.06
M–W: *Z* = 1.02; *p* = 0.31				

*X* arithmetic mean, *SD* standard deviation, *Me* median, *M–W* *U* Mann–Whitney test, *Z* *Z*-value.

The level of FSH in group G1 was statistically significantly higher in patients after orchiectomy than in cases of orchidopexy and in the control group (*p* = 0.01). There were no significant differences in FSH in other puberty groups (*p* > 0.05) (Table 2).

**Table 2:** FSH in the study group (orchidectomy, orchidopexy) and control group—concentration mIU/ml.

Group	Range of values	*X*	SD	Me
Pre-pubertal G1				
Orchidectomy	0.53–2.15	1.00	0.51	0.80
Orchidopexy	0.56–1.83	1.01	0.71	0.64
Control	0.41–0.47	0.45	0.03	0.46
K–W: *H* = 8.46; *p* = 0.01				
Pubertal G4				
Orchidectomy	4.36–8.65	6.51	3.03	6.51
Orchidopexy	0.81–15.34	6.44	5.04	4.62
Control	1.59–8.71	5.15	5.03	5.15
K–W: *H* = 0.09; *p* = 0.96				
Pubertal G5				
Orchidectomy	2.53–10.6	5.95	4.17	4.71
Orchidopexy	1.86–8.57	4.36	2.95	3.51
Control	0.95–3.20	2.06	1.56	2.06
K–W: *H* = 1.78; *p* = 0.41				

*X* arithmetic mean, *SD* standard deviation, *Me* median, *K-W* Kruskal-Wallis test, *H* *H*-value.

Statistical analysis showed no significant differences between the study and control groups in the evaluation of LH, testosterone, oestradiol and VEGF in each puberty group (*p* > 0.05) (Tables 3–6).

**Table 3:** LH in the study group (in general) and control group—concentration mIU/ml.

Group	Range of values	*X*	SD	Me
Pre-pubertal G1				
Study	0.07–0.10	0.09	0.01	0.10
Control	0.10	0.10	0.00	0.10
M–W: *Z* = 0.72; *p* = 0.47				
Pubertal G4				
Study	1.34–7.72	4.03	1.91	3.62
Control	2.60–3.64	3.12	0.74	3.12
M–W: *Z* = −0.59; *p* = 0.56				
Pubertal G5				
Study	2.75–6.64	4.64	1.35	4.92
Control	3.15–4.35	3.75	0.85	3.75
M–W: *Z* = −0.73; *p* = 0.46				

*X* arithmetic mean, *SD* standard deviation, *Me* median, *M–W* *U* Mann–Whitney test, *Z* *Z*-value.

**Table 4:** Testosterone in the study group (in general) and control group—concentration ng/dl.

Group	Range of values	*X*	SD	Me
Pre-pubertal G1				
Study	2.50–3.00	2.90	0.20	3.00
Control	3.00	3.00	0.00	3.00
M–W: *Z* = 0.72; *p* = 0.47				
Pubertal G4				
Study	499.00–847.90	584.27	116.16	524.00
Control	332.00–775.00	553.50	313.25	553.50
M–W: *Z* = −0.12; *p* = 0.91				
Pubertal G5				
Study	219.00–487.30	431.87	172.22	450.00
Control	301.00–366.00	333.50	45.96	333.50
M–W: *Z* = −0.44; *p* = 0.66				

*X* arithmetic mean, *SD* standard deviation, *Me* median, *M*–*W* *U* Mann–Whitney test, *Z* *Z*-value.

**Table 5:** Oestradiol in the study group (in general) and control group—concentration pg/ml.

Group	Range of values	*X*	SD	Me
Pre-pubertal G1				
Study	4.99–30.82	8.87	8.05	4.99
Control	4.99	4.99	0.00	4.99
M–W: *Z* = −0.72; *p* = 0.47				
Pubertal G4				
Study	8.81–55.32	30.19	15.04	27.12
Control	4.99–29.61	17.30	17.41	17.30
M–W: *Z* = −0.82; *p* = 0.41				
Pubertal G5				
Study	16.17–54.23	30.59	14.00	27.19
Control	30.7–38.24	34.47	5.33	34.47
M–W: *Z* = 0.44; *p* = 0.66				

*X* arithmetic mean, *SD* standard deviation, *Me* median, *M*–*W* *U* Mann–Whitney test, *Z* *Z*-value.

**Table 6:** VEGF in the study group (in general) and control group—concentration pg/ml.

Group	Range of values	*X*	SD	Me
Pre-pubertal G1				
Study	98.39–787.15	433.57	235.50	349.53
Control	158.53–324.86	238.01	88.66	234.33
M–W: *Z* = −1.50; *p* = 0.13				
Pubertal G4				
Study	77.87–421.02	229.33	123.24	241.67
Control	110.84–219.03	164.83	76.64	164.83
M–W: Z = −0.59; *p* = 0.56				
Pubertal G5				
Study	79.89–335.43	166.02	106.32	104.01
Control	120.78–310.09	215.44	133.86	215.44
M–W: *Z* = 0.73; *p* = 0.46				

*X* arithmetic mean, *SD* standard deviation, *Me* median, *M*–*W* *U* Mann–Whitney test, *Z* *Z*-value.

Statistical analysis showed no significant differences between cases of orchiectomy, orchidopexy and the control group in evaluation of LH, testosterone, oestradiol and VEGF in each of the puberty groups (*p* > 0.05) (Tables 7–10).

**Table 7:** LH in the study group (orchidectomy, orchidopexy) and control group—concentration mIU/ml.

Group	Range of values	*X*	SD	Me
Pre-pubertal G1				
Orchidectomy	0.07–0.10	0.10	0.01	0.10
Orchidopexy	0.07–0.10	0.08	0.02	0.07
Control	0.10	0.10	0.00	0.10
K–W: *H* = 5.01; *p* = 0.08				
Pubertal G4				
Orchidectomy	1.34–2.94	2.14	1.13	2.14
Orchidopexy	2.69–7.12	4.58	1.76	3.96
Control	2.60–3.64	3.12	0.74	3.12
K–W: *H* = 3.94; *p* = 0.14				
Pubertal G5				
Orchidectomy	3.03–6.64	4.86	1.81	4.92
Orchidopexy	2.75–5.38	4.47	1.18	4.88
Control	3.15–4.35	3.75	0.85	3.75
K–W: *H* = 0.81; *p* = 0.67				

*X* arithmetic mean, *SD* standard deviation, *Me* median, *K*–*W* Kruskal–Wallis test, *H H*-value.

**Table 8:** Testosterone in the study group (orchidectomy, orchidopexy) and control group—concentration ng/dl.

Group	Range of values	*X*	SD	Me
Pre-pubertal G1				
Orchidectomy	2.50–3.00	2.94	0.18	3.00
Orchidopexy	2.50–3.00	2.80	0.26	2.90
Control	3.00	3.00	0.00	3.00
K–W: *H* = 4.55; *p* = 0.10				
Pubertal G4				
Orchidectomy	499.00–847.90	673.45	246.71	673.45
Orchidopexy	503.00–675.40	558.79	66.62	524.00
Control	332.00–775.00	553.50	313.25	553.50
K–W: *H* = 0.09; *p* = 0.96				
Pubertal G5				
Orchidectomy	353.30–476.00	426.43	64.66	450.00
Orchidopexy	219.00–749.00	435.95	237.66	387.90
Control	301.00–366.00	333.50	45.96	333.50
K–W: *H* = 0.44; *p* = 0.81				

*X* arithmetic mean, *SD* standard deviation, *Me* median, *K*–*W* Kruskal–Wallis test, *H H*-value.

**Table 9:** Oestradiol in the study group (orchidectomy, orchidopexy) and control group—concentration pg/ml.

Group	Range of values	*X*	SD	Me
Pre-pubertal G1				
Orchidectomy	4.99–30.82	8.22	9.13	4.99
Orchidopexy	4.99–15.00	10.60	5.11	11.80
Control	4.99	4.99	0.00	4.99
K–W: *H* = 4.15; *p* = 0.13				
Pubertal G4				
Orchidectomy	12.68–24.20	18.44	8.15	18.44
Orchidopexy	8.81–55.32	33.55	15.22	31.76
Control	4.99–29.61	17.30	17.41	17.30
K–W: *H* = 2.92; *p* = 0.23				
Pubertal G5				
Orchidectomy	16.17–54.23	32.53	19.58	27.19
Orchidopexy	19.31–43.88	29.14	11.40	26.69
Control	30.70–38.24	34.47	5.33	34.47
K–W: *H* = 0.34; *p* = 0.84				

*X* arithmetic mean, *SD* standard deviation, *Me* median, *K*–*W* Kruskal–Wallis test, *H H*-value.

**Table 10:** VEGF in the study group (orchidectomy, orchidopexy) and control group—concentration pg/ml.

Group	Range of values	*X*	SD	Me
Pre-pubertal G1				
Orchidectomy	98.39–787.15	441.99	222.26	408.27
Orchidopexy	124.86–758.97	411.12	321.51	349.53
Control	158.53–324.86	238.01	88.66	234.33
K–W: *H* = 2.49; *p* = 0.29				
Pubertal G4				
Orchidectomy	77.87–246.97	162.43	119.58	162.43
Orchidopexy	93.53–421.03	248.45	126.29	241.67
Control	110.64–219.03	164.83	76.64	164.83
K–W: *H* = 1.29; *p* = 0.53				
Pubertal G5				
Orchidectomy	93.45–335.43	177.63	136.76	104.01
Orchidopexy	79.89–299.59	157.31	99.50	124.88
Control	120.78–310.09	215.44	133.86	215.44
K–W: *H* = 0.90; *p* = 0.64				

*X* arithmetic mean, *SD* standard deviation, *Me* median, *K*–*W* Kruskal–Wallis test, *H H*-value.

Studies showed that AMH levels were slightly higher in the control group compared to the study group in pre-pubertal group G1. The observed differences were close to statistical significance (*p* = 0.06) (Table 11).

**Table 11:** AMH in the study group (in general) and control group—concentration ng/ml.

Group	Range of values	*X*	SD	Me
Pre-pubertal G1				
Study	31.30–152.20	79.85	46.08	53.80
Control	100.00–163.00	118.50	29.78	105.50
M–W: *Z* = 1.63; *p* = 0.06				

*X* arithmetic mean, *SD* standard deviation, *Me* median, *M*–*W* *U* Mann–Whitney test, *Z* *Z*-value.

Statistical analysis showed no correlation between testosterone concentration and TTV in pubertal study groups G4 and G5 (Table 12).

**Table 12:** Spearman’s (*R*) correlation between testosterone concentration (ng/dl) and total testicular volume (ml) in pubertal study groups.

Testosterone concentration				Total testicular volume			
Range of values	*X*	SD	Me	Range of values	*X*	SD	Me
Pubertal G4							
499.00–847.90	584.27	116.16	524.00	13.20–27.10	21.08	5.08	23
*R* = −0.15, *p* = 0.70							
Pubertal G5							
219.00–487.30	431.87	172.22	450.00	14.20–40.00	23.75	8.62	20
*R* = −0.46, *p* = 0.29							

*X* arithmetic mean, *SD* standard deviation, *Me* median.

## Discussion

Conducting our research, we tried to answer the question “What are the risks for men who suffered from testicular torsion during the developmental period?”. One of them is the theory about specific antibodies against sperm and testicular antigens formed due to TT in the serum and semen that impair fertility.^2,5,7,9,14–17^

The results of studies on antibodies in patients after TT described in the literature are divergent. The occurrence of antibodies against sperm in serum was reported by Yang et al.,^2^ Rybkiewicz et al.^7^ and Fu et al.,^17^ while their presence in sperm was described by Arap et al.,^5^ Anderson and Williamson,^12^ Hagen et al.^13^ and Mastrogiacomo et al.^14^ Arap et al.^5^ indicated elevated titres of antibodies in the study groups (orchiectomy and orchidopexy), but without significant differences between groups. Neither do they describe the relationship between the titre of antibodies and the age at which torsion occurred nor its duration. The studies of Fraser et al.^27^ and Puri et al.^28^ did not show the presence of these antibodies.

Anderson and Williamson^12^ did not detect the presence of antibodies against testicular antigens in the serum. Zanchetta^15^ described antibodies against Leydig cells in a few percent of patients after TT.

No antibodies against sperm or Leydig cells were found in the serum in our study group or the control group. Yang et al.^2^ and Arap et al.^5^ have been observing patients for the presence of antibodies for 5–10 years. In our material, the average observation period was 3 years. We believe that this was sufficient time for the development of an autoimmune response, both humoral and cellular. Of course our patients could developed antibodies only in the semen and they were sero-negative.

When discussing the role of VEGF, it is worth noting that the lack of even one allele encoding VEGF causes the death of the foetus.^29,30^ VEGF is a factor whose secretion is strongly stimulated in ischaemic tissues, and its elevated levels are found in many autoimmune processes.^21,22^ VEGF has been shown to be crucial for the sex-specific vascularisation of the testis.^23^ We know from the literature that both relay pathway inhibitors and excess isoforms for VEGF cause vascular development disorders in the testis and in the formation of the spermatic cord.^23,31^ An excess of VEGF causes infertility as a result of spermatogenesis retention and sex epithelium hypertrophy.^31^ However, mouse studies have shown that VEGF is not absolutely necessary for normal testicular development, although the development of vessels in this case may be delayed in the foetal period.^25^ This does not diminish the importance of VEGF for testicular function and fertility. In the study by Lu et al.^25^, the loss of VEGF isoforms in mice resulted in reduced sperm abundance and altered expression of genes that regulate undifferentiated spermatogonium. It also resulted in lower body weight, and a smaller epididymis and prostate. In the case of TT, in the mechanism of tissue hypoxia, we should deal with an increase in the concentration of VEGF the next after torsion. Co-existing autoimmune process should also result in increased concentration of this growth factor.

Taking into account the role of VEGF in the proper development and functioning of testes and its elevated level in the serum in autoimmune processes, which has been widely described in the literature, we have evaluated the remote influence of TT on the secretion of that growth factor. We found that its concentrations in the study and control groups of our patients were comparable.

Summarising the above considerations, it should be stated that the issue of the presence of specific antibodies and rise in VEGF levels in boys after an episode of TT require further research.

Studies of hormones in our patients allowed us to refer to the theory of primary dysplasia.^5,11–13^ The studies performed so far have shown that the concentration of gonadotropins was higher but within the limits of the norm in men who had TT, which caused damage to the testis or the necessity for orchiectomy, in comparison with healthy men.^5,18^ A study by Arap et al.^5^ shows that FSH concentrations are higher in patients who have undergone TT than in the control group, and patients treated with orchiectomy had higher gonadotropin concentrations than those treated with detorsion. Another study evaluating gonadotropin concentrations was the work of Romero et al.^18^ They measured gonadotropin concentrations before and after gonadoliberine stimulation; the values obtained were normal in all subjects and higher in the group treated with orchiectomy than with detorsion, but they were not compared to the control group. This study also shows that patients have lower concentrations of inhibin B after an episode of TT, which is considered to be a meaningful quantitative index of the function of Sertoli cells. The longer the duration of anoxia is, the greater the potential loss of functional weight of the testis and thus the number of Sertoli cells.^32^ This results in the reduced release of factors, such as inhibin B, from the testis. This process is responsible for the increased secretion of FSH.^33^ The increased secretory function of the pituitary gland is an indirect indicator of functional loss of testicular tissue.^34^

In our study, FSH concentrations were within normal limits in most cases; however, they were statistically significantly higher in the pre-pubertal G1 group compared to the control group. Analysing the G1 group, it was found that the concentration of FSH was the highest in patients whose testes were removed, lower in which they were preserved, and the lowest in the control group. In the remaining groups (G4 and G5), these concentrations were higher than in the control group; however, they did not show statistical significance. This is indirect proof of the lower mass of active testicular tissue, but it does not explain whether the lower mass is a secondary effect to the damage or a primary abnormality.

LH secreted from the pituitary gland regulates the function of Leydig cells producing testicular androgens, especially testosterone. Animal studies indicate that testicular ischaemia or poorer function of Leydig cells may adversely affect pulsed testosterone secretion.^35^ In the study of Arap et al.^5^ higher concentrations of LH were found in patients treated with orchiectomy; however, similar to the study of Romero et al.^18^, these concentrations were within the limits of the norm.

When evaluating LH concentration in our material, we did not show statistically significant differences between the study group and the control group.

Studies carried out on rats showed that TT impairs testicular steroidogenesis.^19,20^ Turner et al.^20^ examined the concentration of testosterone in serum from the testicular vein. Experimental TT significantly reduced its concentration 3 and 30 days after the detorsion. Testosterone concentration was inversely proportional to reperfusion indexes after the operation on the twisted testis. In the study by Kurt et al.^19^ testosterone concentrations measured 3 and 30 days after the surgical intervention depended on the duration of torsion.^19^ All rats had their left testis removed, and groups were distinguished depending on the duration of the torsion (1, 3 and 5 h, respectively). In the control group, the testis was not twisted. Testosterone concentrations in all groups were statistically significantly lower than in the control group on the third day after torsion. On the 30th day after the operation, an increase in testosterone concentration was observed in all groups. Only in the group of rats in which the torsion lasted for 5 h testosterone concentrations were significantly lower than in the control group. Our study showed that testosterone levels in patients after TT did not differ from the standards for age and puberty and were also comparable to the control group. Similar results were obtained by Arap et al.^5^ Romero et al.^18^ obtained a significant correlation between the amount of inhibin B secreted, testosterone concentration and TTV. Our study did not show any effect of TTV on testosterone secretion in pubertal groups G4 and G5. In our opinion, one testis has enough Leydig cells and is able to compensate for the damage to the second testis, so we believe that patients after TT are not at risk from symptoms of hypogonadism due to testosterone deficiency.

AMH is a compound secreted by Sertoli cells. Its basic secretion is independent of gonadotropins or sex steroids, but FSH increases and testosterone inhibits AMH secretion.^36^ It plays a key role in the differentiation of external and internal genital organs, by blocking the development of Müller’s ducts and consequently causing their atrophy, thereby preventing the development of female genital organs.^32^ It is also very important for the correct course of the testicular descent process. Low AMH values may reflect primary testicular dysfunction, (e.g., in patients with cryptorchidism or partial gonadal dysgenesis).^36^ Serum AMH levels are highest in the pre-pubertal period.^36^

In our study, AMH levels were statistically significantly higher in the control group than in the pre-pubertal G1 group. However, it is worth noting that several boys in the pre-pubertal group had significantly lower AMH concentrations than other boys. Serum concentrations in five boys aged 2 to 7 years ranged from 31.3 to 50 ng/ml (testes were removed in four boys, while the testis was preserved in one), while they were >100 ng/ml in the control group. Moreover it is important that in four patients who had testes removed in this group, AMH concentrations were comparable to the control group. This may indicate a certain dysfunction of gonads in patients with lower AMH concentrations. It is possible that it has its primary character.

The primary dysplasia, which in combination with anatomical disorders of the testicular region may predispose to its torsion, is described in Selami Sozubir’s experimental study.^37^ He determined the influence of insuline-like factor 3 (INSL3) on the descending process of testes, their mobility, macroscopic structure, as well as the influence on the spermatic cord and its predisposition to torsion. In this study, three groups of mice with different expression of the gene responsible for the production of INSL3 were identified: INSL3 wild type (INSL3+/+), heterozygous (INSL3+/−) and knockout (INSL−/−). INSL3+/+ mice had correctly descended testes and formed spermatic cords. In the case of INSL3−/− mice had bilateral cryptorchidism, incorrectly formed and often twisted spermatic cords. INSL3+/− mice had descended testes or cryptorchidism, the structure of spermatic cords was better than in INSL3 knockout ones, but they were often twisted. TT occurred in a part of mice. There are many papers on cryptorchidism and hormonal factors. The Sozubir’s study suggests that the same factors are involved in cryptorchidism and TT, but in the case of TT this has not been so well documented. In terms of dysgenesis, these two disease entities are based on an abnormal process of descending and anchoring the testes in the scrotum. In addition to primary dysplasia, it should be mentioned of the so-called endocrinological disruptors, environmental pollution and food pollution, which have oestrogenic activity. They may favour TT, but this requires further research.

However oestradiol is a hormone responsible for normal spermatogenesis, it reduces the volume of spermatogenic tubules and impairs testicular function when present in excess.^38,39^ If there is an excess of endogenous oestradiol, it can also affect the occurrence of TT, especially in the youngest age groups. Oestradiol levels in our patients from the study group did not differ from the control group.

In the case of G1 boys with reduced AMH levels, the cause of TT may be the same as for cryptorchidism. However, the diversity of the G1 group seems to confirm the multitude of potential predisposing and causative factors among younger boys. In the older groups, G4 and G5, higher FSH concentrations seem to be rather due to damage to testicular tissue. In these patients, too, could the primary dysplasia have affected the occurrence of TT? We cannot go back to the past and check their hormone profiles in the first years of life. However, indirectly, looking at the compensation for testosterone concentrations, this seems to contradict the high gonadal dysfunction in these patients. Patients with severe hypogonadism are unable to produce enough testosterone, even though two testes are present. A broader and unambiguous picture would be whether there is impaired fertility of these men as a result of TT. In the case of normal fertility, the theory of gonadal dysplasia in TT at an older age could be rejected.

To sum up, the fertility disorders in patients after TT should be mentioned. Subfertility generally describes any form of reduced fertility with prolonged time of unwanted non-conception.^40^

According to Arap et al.^5^, there are no statistically significant differences in sperm count and sperm mobility between a group of patients after orchiectomy and a group after detorsion of the testis due to TT and a control group. In none of the groups the sperm had an average normal morphology according to WHO criteria. The Puri’s publication describes correct results of sperm analysis only in 77% of men after TT in terms of sperm volume, number and mobility of spermatozoa.^28^ Jacobsen et al.^41^ present a meta-analysis of studies on the sperm in patients treated for TT, concluding that the mobility of spermatozoa is decreasing in most of them and that the number of spermatozoa is falling below 20 mln/ml. This fulfils the conditions of the WHO concept of “subfertile”.

The problem of fertility in terms of pregnancy is discussed in two publications. According to Mäkelä’s study,^42^ children were born in relationships to 56% of men after an orchiectomy because of TT and as many as 81% of men who had a testicular detorsion. Zhang et al.,^43^ on the other hand, analysed the percentage of children born by comparing the type of operation in the case of testicular torsion (relationship: orchiectomy/detorsion) and the age at which the man was operated on. They stated that the most favourable dependence (93%/100%) was in cases of operations under 14 years old .

## Conclusions

Results of our study did not confirm the autoimmune process in boys operated on due to TT. The pituitary–testis axis seems to have sufficient compensation capabilities. However, study results suggest that primary gonadal dysplasia may predispose to testicular torsion.
